# Systemic inflammation and causal risk for Alzheimer's dementia: Possibilities and limitations of a Mendelian randomization approach

**DOI:** 10.1002/agm2.12046

**Published:** 2018-12-05

**Authors:** Alex Tsui, Daniel Davis

**Affiliations:** ^1^ MRC Unit for Lifelong Health and Ageing at UCL London UK

## Abstract

Epidemiological studies have implicated systemic inflammation in the development of Alzheimer's disease (AD). However, these observations have been subject to residual confounding and reverse causation. We applied Mendelian randomization approaches to address this. We did not identify any causal associations between serum interleukin (IL)‐18, IL‐1ra, IL‐6, or erythrocyte sedimentation rate (ESR) concentrations and AD. Our findings are limited by the low number of available instruments, though some of those identified (e.g., IL‐6) were of sufficient power to indicate true negative results. Taken together, it appears there is no evidence for a causal association between these serum inflammatory cytokines and AD.

## 1 INTRODUCTION

Systemic inflammation has been linked to chronic neurodegenerative conditions, such as Alzheimer's disease (AD), in multiple contexts.^1^ In epidemiological studies, chronic systemic inflammation has been prospectively associated with smaller brain volumes and poorer episodic memory in later life.^2^ In persons living with AD, systemic inflammation—both chronically at baseline and through acute episodes—predicted increased rates of cognitive decline.^3^ For individuals without a diagnosis of dementia, episodes of critical illnesses have been associated with cognitive decline in later life.^4^ In population samples, individuals reporting symptoms of delirium have shown reduced cognitive performance at long‐term follow‐up.^5^, ^6^

A number of risk factors for AD, such as educational attainment, physical activity, smoking, hypertension, obesity, diabetes, and depression, have been identified in large population‐based studies.^7^ Some of these associations may operate via systemic inflammation, though these observations may also be affected by residual confounding. Therefore, it remains uncertain whether systemic inflammation directly contributes to Alzheimer's pathology, or reflects the contributions of other modifiable risk factors to AD. Yet, understanding if there are causal mechanisms between systemic inflammation and dementia would be vital if this presented a novel therapeutic target.

Mendelian randomization (MR) overcomes a number of limitations when assessing causation from observational data.^8^ Using random variations in genes of known function to study the causal effect of a modifiable exposure on a disease outcome, the susceptibility to confounding is reduced. In addition, the occurrence of genetic allocation at the time of conception eliminates the likelihood of reverse causation. Although two recent MR studies have demonstrated no causal association between C‐reactive protein (CRP), a broad marker of systemic inflammation, and dementia diagnosis,^9^, ^10^ the potential causative associations between serum inflammatory cytokines and dementia diagnosis remain unknown. In this study, we first aimed to identify serum inflammatory cytokines with genetic instruments in current literature, and second, explore whether a causal association exists between individual serum inflammatory markers and AD diagnosis using a two‐sample MR technique,^11^ which harnesses summary data available in literature from non‐overlapping datasets and is less susceptible to weak‐instrument bias observed in one‐sample MR.^12^

## 2 METHODS

The International Genomics of Alzheimer's Project (IGAP) is a large two‐stage study based upon genome‐wide association studies (GWAS) on individuals of European ancestry. In stage 1, IGAP used genotyped and imputed data on 7 055 881 single nucleotide polymorphisms (SNPs) to meta‐analyze four previously published GWAS datasets consisting of 17 008 AD cases and 37 154 controls (the European Alzheimer's Disease Initiative [EADI], the Alzheimer Disease Genetics Consortium [ADGC], the Cohorts for Heart and Aging Research in Genomic Epidemiology Consortium [CHARGE], and the Genetic and Environmental Risk in AD Consortium [GERAD]). In stage 2, 11 632 SNPs were genotyped and tested for association in an independent set of 8572 AD cases and 11 312 controls. Finally, a meta‐analysis was performed combining results from Stages 1 and 2.

### 2.1 Selecting genetic instruments for inflammation biomarkers

SNPs for inflammation biomarkers were identified using the NHGRI‐EBI catalog of published GWAS studies (https://www.ebi.ac.uk/gwas/home). GWAS studies of individuals of European descent were further examined, though we could only consider SNPs for which complete data on minor allele frequency, beta coefficients, standard errors (SE), and *P* values were available. The catalogue search identified 22 SNPs from three eligible studies for IL‐18,^13^, ^14^ seven SNPs from two studies for IL1‐receptor antagonist (IL‐1ra),^13^, ^15^ 16 SNPs from two studies for IL‐6,^16^, ^17^ and four SNPs from one study for erythrocyte sedimentation rate (ESR).^18^

For all SNPs, a *P* value association threshold of 5 × 10^−8^ was applied for Bonferroni correction and a minor allele frequency >0.3. Where linkage disequilibrium amongst clumped SNPs was >0.2, the SNP with greatest beta coefficient was chosen as the index. When SNPs from the two samples were matched, one SNP for IL‐18 (rs17229943), one for IL‐6 (rs28638007), and one for ESR (rs11829037) did not correspond. A suitable proxy SNP with linkage disequilibrium of >0.8 could not be identified and these SNPs were excluded from further analysis. Nine SNPs were palindromic (rs657152, rs4537545, rs7529229, rs4129267, rs4553185, rs4845618, rs4845625, rs4845371, and rs12740969), resulting in potential strand ambiguity. Allele frequencies for these were compared between the inflammatory markers and IGAP datasets to ensure that effect estimates were recorded with respect to the same effect allele. The proportion of total variance explained for each independent was calculated by the following formula:

$$\text{Variance explained}={\left(\beta \times \sqrt{2\times \text{MAF}(1-\text{MAF})}\right)}^{2},$$

where MAF = minor allele frequency. For the two‐sample MR analysis, the number of index SNPs used for IL‐18, IL‐1ra, IL‐6, and ESR was 2, 3, 3, and 4, respectively.

### 2.2 Instrumental variables analyses in summary data

Two‐sample MR was used to estimate the causal associations of each exposure (IL‐18, IL‐1ra, IL‐6, and ESR)^13^, ^15^, ^16^, ^17^, ^18^ on the outcome (AD diagnosis). First, inverse variance‐weighted (IVW) regression was used to provide a combined estimate of the causal estimates (SNP‐Alzheimer's/SNP‐biomarker) from each SNP, equivalent to a two‐stage least squares analysis using individual‐level data. All *P* values were two tailed. Second, in order to account for potential horizontal pleiotropy, MR‐Egger was performed if three or more index SNPs were available for the biomarker, introducing an additional parameter for unbalanced pleiotropy. In MR‐Egger, linear regression of the SNP‐Alzheimer's effect is performed on SNP‐biomarker effect, where the slope represents the coefficient of the causal effect while the intercept represents the net bias attributable to horizontal pleiotropy. MR‐Egger analyses assumed that individual SNP effects on the outcome were independent of their pleiotropic effects (InSIDE assumption). All MR analyses methods should yield similar results in the absence of horizontal pleiotropy. All MR‐Egger analyses were performed using the mregger package in Stata version 14.

## 3 RESULTS

### 3.1 Studies and participants

Genome‐wide association data for inflammation markers were included from six meta‐analyses or prospective studies with participants of White Caucasian ancestry. GWAS data for IL‐18 included 12 736 participants (3233 from Cardiovascular Health Study, 1210 from InCHIANTI study, and 8293 from Finrisk and Cardiovascular Health in Young Finns study); 12 381 participants for IL‐1ra (3233 from Cardiovascular Health Study, 1210 from InCHIANTI study, 7938 from Whitehall II Study); 14 501 participants for IL‐6 (4911 Whitehall II, 3445 British Women's Heart and Health Study, and 6145 from the SarDINIA Study); and data on 6145 participants were available for ESR. The variance in biomarker levels explained by selected genetic instruments was 3.15%, 1.95%, 3.23%, and 2.99% for IL‐18, IL‐1ra, IL‐6, and ESR, respectively.

### 3.2 MR analysis

The causal odds ratios (OR) for the association between AD and individual inflammation markers are shown in Table 1. Using IVW regression, IL‐18, IL‐1ra, and IL‐6 did not appear to be causally associated with AD. Although IVW suggested a significant association for ESR and AD, this estimate was attenuated when horizontal pleiotropy was taken into account, similar to the MR‐Egger analyses for IL‐1ra and IL‐6. The degree of horizontal pleiotropy could not be tested for IL‐18 due to the lack of instrumental variables.

**Table 1:** SNPs identified for IL‐18, IL‐1‐ra, IL‐6, and ESR from current literature

Biomarker	Chromosome	Position	SNP	IGAP effect allele	Biomarker effect allele	Minor allele frequency	Variance explained (%)
IL‐18	2	32489851	rs385076	C	C	0.34	2.65
IL‐18	11	112085316	rs2250417	T	T	0.48	0.50
IL‐1ra	2	113834820	rs13386602	A	A	0.44	0.83
IL‐1ra	2	113832333	rs6743376	A	A	0.43	0.83
IL‐1ra	2	113874467	rs4251961	T	T	0.32	0.29
IL‐6	14	52083080	rs1008924	A	A	0.507	0.62
IL‐6	1	154426264	rs4129267	T	A	0.41	0.31
IL‐6	9	136142355	rs643434	G	G	0.742	2.30
ESR	1	207803021	rs11803956	T	T	0.752	0.88
ESR	16	78019687	rs11861089	C	T	0.694	0.57
ESR	1	207739127	rs3886100	A	A	0.772	0.89
ESR	11	5306509	rs4910742	G	A	0.934	0.65

ESR, erythrocyte sedimentation rate; IGAP, International Genomics of Alzheimer's Project; IL, interleukin; SNPs, single nucleotide polymorphisms.

## 4 DISCUSSION

In our study, no causal associations were found between serum IL‐18, IL‐1ra, IL‐6, or ESR concentrations and AD. The number of valid, independent genetic instruments for serum inflammatory cytokines available in current published literature is limited. Overall, our findings do not support a causal role for inflammation on risk of Alzheimer's dementia (see Table 2).

**Table 2:** Mendelian randomization results for IL‐18, IL‐1ra, IL‐6, and ESR in Alzheimer's disease

Biomarker	No. SNPs	Beta	95% CI		*P* value
IL‐18					
IVW	2	0.96	0.18	5.10	0.82
IL‐1ra					
IVW	3	1.02	0.64	1.49	0.85
MR‐Egger		1.04	0.25	4.27	0.96
IL‐6					
IVW	3	1.00	0.96	1.03	0.78
MR‐Egger		1.02	0.76	1.26	0.87
ESR					
IVW	4	1.37	1.01	1.85	0.05
MR‐Egger		1.62	0.39	6.63	0.50

CI, confidence interval; ESR, erythrocyte sedimentation rate; IL, interleukin; IVW, inverse variance‐weighted; MR, Mendelian randomization; SNPs, single nucleotide polymorphisms.

A strength of our approach was to use Mendelian randomization to investigate causal associations between serum inflammatory cytokines and diagnosis of AD, minimizing confounding and reverse causation observed in traditional epidemiological studies. We utilized the largest available genetic database for AD, with over 17 000 cases from over 50 000 samples.^19^ This study is also sufficiently powered for causal associations between IL‐6 and Alzheimer's dementia, and likely powered for IL‐18 and AD as suggested by power calculations using concentrations of cytokines reported in the literature (http://cnsgenomics.com/shiny/mRnd).^20^ These suggest that for power of 0.8 and type 1 error rate of 0.05, minimum sample sizes required for IL‐6 were 727 to 5137,^21^, ^22^ and from 3657 to 26 678 for IL‐18.^23^ Power calculations were not possible for ESR or IL‐1ra due to a lack of comparative serum levels in the literature between individuals with and without AD.^24^ The main weakness of this study was the small number of genetic instruments for serum cytokines available in the current literature, limiting proportion of variance explained and assessments of horizontal pleiotropy. Second, our findings pertain only to people of European ancestry.

Is there scope for future studies investigating systemic inflammation and AD? Certainly further genetic associations may become evident if yet larger cohorts can establish stronger instruments for serum inflammatory cytokines. One approach would be multivariate GWAS within a single sample used to additionally describe SNP effects on several serum cytokines.^25^ In addition, the precision of AD diagnosis can be improved with better phenotyping, even with simple imaging. On current evidence, however, it appears that causative associations between serum inflammation and AD cannot be demonstrated using this approach.

## CONFLICT OF INTEREST

The authors have no conflict of interest to report.
